# Biochemical Characterization of a New β-Agarase from *Cellulophaga algicola*

**DOI:** 10.3390/ijms20092143

**Published:** 2019-04-30

**Authors:** Zhenggang Han, Yuxi Zhang, Jiangke Yang

**Affiliations:** College of Biology and Pharmaceutical Engineering, Wuhan Polytechnic University, Wuhan 430023, China; zhengganghan@whpu.edu.cn (Z.H.); zhangyuxi162@163.com (Y.Z.)

**Keywords:** agar, agarose, β-agarase, glycoside hydrolase, neoagarooligosaccharide

## Abstract

*Cellulophaga algicola* DSM 14237, isolated from the Eastern Antarctic coastal zone, was found to be able to hydrolyze several types of polysaccharide materials. In this study, a predicted β-agarase (*Ca*Aga1) from *C. algicola* was heterologously expressed in *Escherichia coli*. The purified recombinant *Ca*Aga1 showed specific activities of 29.39, 20.20, 14.12, and 8.99 U/mg toward agarose, pure agar, and crude agars from *Gracilaria lemaneiformis* and *Porphyra haitanensis*, respectively. *Ca*Aga1 exhibited an optimal temperature and pH of 40 °C and 7, respectively. *Ca*Aga1 was stable over a wide pH range from 4 to 11. The recombinant enzyme showed an unusual thermostability, that is, it was stable at temperature below or equal to 40 °C and around 70 °C, but was thermolabile at about 50 °C. With the agarose as the substrate, the *K_m_* and *V_max_* values for *Ca*Aga1 were 1.19 mg/mL and 36.21 U/mg, respectively. The reducing reagent (dithiothreitol) enhanced the activity of *Ca*Aga1 by more than one fold. In addition, *Ca*Aga1 was salt-tolerant given that it retained approximately 70% of the maximum activity in the presence of 2 M NaCl. The thin layer chromatography results indicated that *Ca*Aga1 is an endo-type β-agarase and efficiently hydrolyzed agarose into neoagarotetraose (NA4) and neoagarohexaose (NA6). A structural model of *Ca*Aga1 in complex with neoagarooctaose (NA8) was built by homology modeling and explained the hydrolysis pattern of *Ca*Aga1.

## 1. Introduction

Agar is an algal galactan extracted from marine red algae (*Rhodophyta*), such as *Gelidium*, *Gracilaria*, and *Porphyra* [1,2]. The backbone structure of agar generally consists of alternating 3-O-β-D-galactose and 4-O-α-L-galactose [3]. Agars derived from different red algal species have variable chemical structures. A significant proportion of α-L-galactose residues are present in derivatized forms with substitutions of sulfate esters, methyl esters, and pyruvate acetals [4]. A linear galactan comprised of repetitive disaccharide units of β-D-galactose (G)/3,6-anhydro-α-L-galactose (L-AHG) is termed agarose [3]. Agarose constitutes the main fractions of agars from the *Geldium* and *Gracillaria* sp. [3,4]. The agar material, extracted from *Porphyra* sp. is called porphyrin, in which significant amount of α-L-galactose residues exist as α-L-galactopyranose-6-sulfate (L6S) [5].

Agar has great economic importance as a functional food ingredient, a nutraceutical, and a microbial growth media due to its stabilizing and gelling properties [3]. Agar polysaccharides are potential alternatives to cellulose as the raw material for preparing fermentable monosaccharides due to their comparable abundance in nature [6,7]. Agar oligosaccharides, obtained by the enzymatic degradation of agar materials, exhibit unique physiological activities and are thus used in the fields of health food, cosmetic, and pharmaceutics [3,4].

Biodegradation of agar is catalyzed by α-agarases (E.C.3.2.1.158) and β-agarases (EC 3.2.1.81), which show different cleavage patterns [8]. Agarooligosaccharides (AOSs) and neoagarooligosaccharides (NAOSs) are the main enzymatic products of agarases, with L-AHG and D-galactose at the reducing ends, respectively. AOSs are obtained through agarose hydrolysis using α-agarases which cleave α-1,3-glycosidic bonds; meanwhile, NAOSs are the hydrolysates of β-agarases, which specifically act on β-1,4-glycosidic bonds [4]. AOSs and NAOSs exert many physiological activities, such as prebiotics [9], skin whitening [10], anti-oxidants [11], and anti-inflammation [12]. Moreover, agarases can be used to liberate nucleic acids from agarose gels [13] and prepare seaweed protoplasts [14].

To date, a great number of agarases, most of which belong to β-agarases, are characterized in marine bacteria and human gut microbiota [2]. According to the Carbohydrate-Active enzymes Database (CAZy, http://www.cazy.org/), α-agarases belong to the glycoside hydrolase (GH) family 96 (GH96) and β-agarases are mainly categorized into GH16, 50 GH50, GH86, and GH118 [3]. The demand for agarolytic enzymes with excellent activity and desirable biochemical properties has increased to meet the requirements for industrial applications. For example, good thermostability is required to prevent gelation of agar materials during hydrolysis [15].

Currently, there are vast genomic sequences available in the public database (such as GenBank). To mine the enzyme resources from genomes of marine microorganisms is an effective approach to discover new agarases. The marine microorganism *Cellulophaga algicola* DSM 14237, isolated from the Eastern Antarctic coastal zone, was found to be able to hydrolyze a wide range of polysaccharides (agar, starch, gelatine, and carboxymethylcellulose) [16]. Genomic sequencing revealed that the bacterium encodes three putative agarases [16]. This study reported the biochemical characterization of *Ca*Aga1, one of the agarases from *C. algicola* DSM 14237. The recombinant *Ca*Aga1 was active towards agarose, pure agar, and crude agar extracted from red algae. The enzyme was active and stable over wide temperature and pH ranges. The biochemical characteristics of *Ca*Aga1 indicated the great potential for application in many industrial processes.

## 2. Results

### 2.1. Amino Acid Sequence Analysis of CaAga1

**Figure 1:**
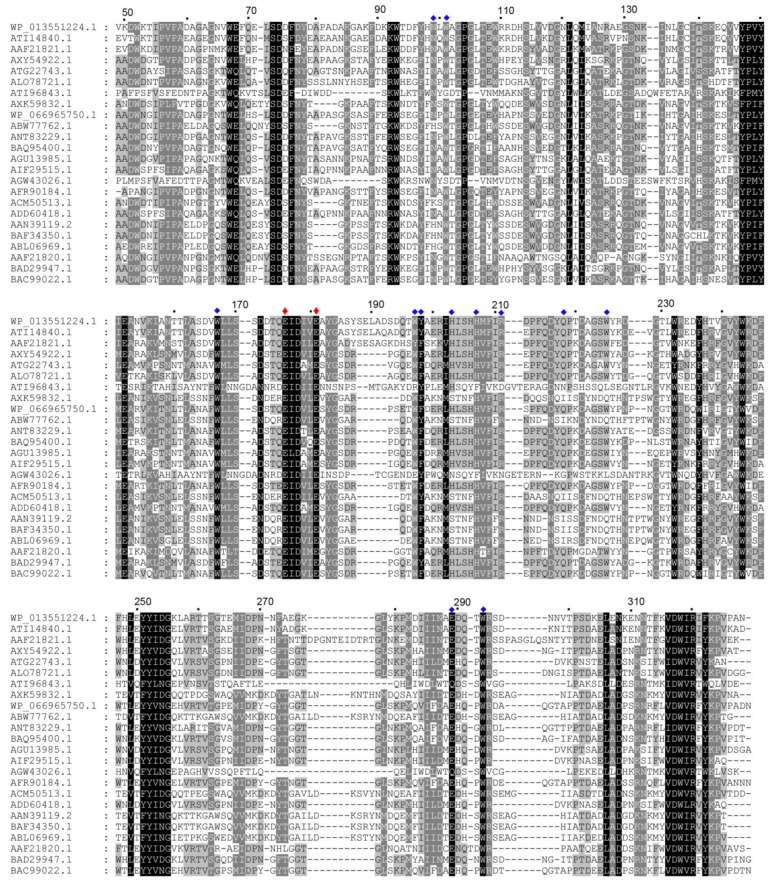
Multiple sequence alignment of GH16 domains of the characterized β-agarases. The alignment of amino acid sequences was carried out using Clustal Omega. The conserved and semi-conserved amino acids are shown in black and gray backgrounds, respectively. The amino acid numbering refers to the sequence of *Ca*Aga1. GenBank accession numbers—WP_013551224.1 for *Ca*Aga1 from *Cellulophaga algicola* DSM 14237, ATI14840.1 for Aga2 from *Cellulophaga omnivescoria* W5C, AAF21821.1 for *Zg*AgaB from *Zobellia galactanivorans*, AXY54922.1 for N3-1 from *Microbulbifer* sp. BN3, ATG22743.1 for Aga0917 from *Pseudoalteromonas fuliginea* YTW1-15-1, ALO78721.1 for Gaa16A from *Gilvimarinus agarilyticus* JEA5, ATI96843.1 for YM01-1 from marine bacterium *Catenovulum agarivorans* YM01, AXK59832.1 for Aga862 from *Pseudoalteromonas* sp. Q30F, WP_066965750.1 for β-agarase from *Microbulbifer* sp. Q7, ABW77762.1 for β-agarase from *Agarivorans albus*, ANT83229.1 for AgaML from a mangrove soil metagenomic library, BAQ95400.1 for AgaTM2 from *Simiduia* sp. TM-2, AGU13985.1 for YM01-3 from *Catenovulum agarivorans* YM01^T^, AIF29515.1 for AgaH71 from *Pseudoalteromonas hodoensis*, AGW43026.1 for AgaG1 from *Alteromonas* sp. GNUM-1, AFR90184.1 for Agy1 from *Saccharophagus* sp. AG21, ACM50513.1 for AgaA from *Agarivorans* sp. LQ48, ADD60418.1 for AgaP from *Pseudoalteromonas* sp. AG4, AAN39119.2 for AgaA from *Pseudoalteromonas* sp., BAF34350.1 for AgaD from *Vibrio* sp. strain PO-303, ABL06969.1 for AgaV from *Vibrio* sp. strain V134, AAF21820.1 for *Zg*AgaA from *Zobellia galactanivorans*, BAD29947.1 for AgaA from the marine isolate JAMB-A94, and BAC99022.1 for AgaA7 from a novel species of deep-sea *Microbulbifer*. The putative catalytic amino acids (nucleophile and acid/base catalyst) and amino acid residues involved in the agarose chain binding are indicated by red and blue diamonds, respectively.

*Ca*Aga1 (GenBank accession number: WP_013551224.1) is the putative agarase annotated in the genome of *C. algicola* DSM 14237. The protein has 328 amino acids and a theoretical molecular mass of 3,7510.57 Da. A signal peptide of 21 amino acids is present in the amino terminal of the protein, as predicted using the SignalP-5.0 Server (*D* = 0.581, *D*-cutoff = 0.450). The similarity analysis of the amino acid sequences conducted using BLASTp search indicated that *Ca*Aga1 is a β-agarase belonging to GH16 family and it shows a high identity (76%) to β-agarase (Aga2) from agarolytic bacteria *Cellulophaga omnivescoria* W5C (GenBank accession number: ATI14840.1) (Figure 1) [17]. *Ca*Aga1 shares 38–76% sequence identities with reported GH16 β-agarases (Figure 1). Multiple sequence alignment revealed that the strictly conserved catalytic motif, ExDxxE (Glu176, Asp178, and Glu181), is present in *Ca*Aga1. Glu176 and Glu181 were supposed to be the nucleophile and the acid/base catalyst in *Ca*Aga1, respectively. The amino acid residues that were proposed to be involved in agarose chain binding were largely conserved in *Ca*Aga1 (Figure 1).

### 2.2. Structural Characteristic of CaAga1

**Figure 2:**
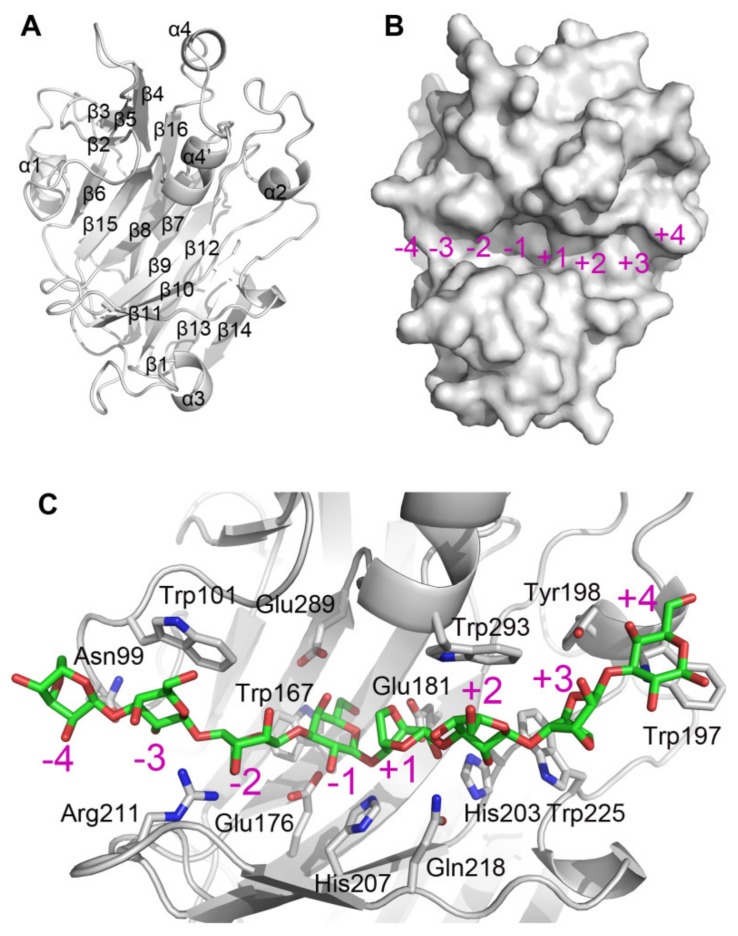
Structural model of *Ca*Aga1 and the interaction detail between *Ca*Aga1 and the agarose chain. (**A**) A ribbon presentation of *Ca*Aga1 with labeled secondary structural elements. (**B**) A surface presentation of *Ca*Aga1. The number and location of subsites in the substrate-binding cleft are shown. (**C**) A model of *Ca*Aga1 in complex with neoagarooctaose (NA8) obtained by superposing the structures of *Ca*Aga1 and the *Zg*AgaB-NA8 complex (PDB entry: 4ATF). The amino acid residues participating in the interaction with the agarose chain are presented in stick. The carbon atoms in NA8 and amino acid residues from *Ca*Aga1 are shown in green and gray, respectively.

A structural model of *Ca*Aga1 was generated by homology modeling using the crystal structure of *Zg*AgaB from *Zobellia galactanivorans* (PDB entry: 1O4Z) as a template (amino acid sequence identity: 68%). As shown in Figure 2A, *Ca*Aga1 has a typical β-jelly roll fold. A number of β-strands (β6, β15, β8, β9, β10, and β11) formed a long and narrow concavity on the protein surface, which is the putative substrate-binding cleft of agarase (Figure 2B). The nucleophile (Glu176) and the acid/base catalyst (Glu181) are located in the middle of the cleft (Figure 2B). A structural model of *Ca*Aga1 in complex with neoagarooctaose (NA8) was built by superposing the structures of *Ca*Aga1 and the *Zg*AgaB-NA8 complex (PDB entry: 4ATF). The resulting model indicated that the substrate-binding cleft could be divided into eight regions, corresponding to eight sugar-binding subsites (−4 to +4) (Figure 2B). Amino acid residues, namely, Asn99, Trp101, Trp167, Trp197, Tyr198, His203, His207, Arg211, Glu218, Trp225, Glu289, and Trp293, and catalytic residues (Glu176 and Glu181), interacted with the agarose chain through hydrophobic stacking or hydrogen bond (Figure 2C). The amino acids within the vicinity of the catalytic residues were conserved among GH16 agarases except a few in the distal region of the cleft (Figure 1).

No additional polysaccharide-binding site (presented in *Zg*AgaA from *Z. galactanivorans* and proposed to unwind the double-helical structure of agarose) was observed in the rear side of the substrate-binding cleft in *Ca*Aga1. Amino acid residues involved in sugar binding (in the additional polysaccharide-binding site) in *Zg*AgaA (Trp87, Asn89, Gln92, Asp271, and Trp277) appeared to be Leu115, Val117, Asn120, Lys309, and Lys315 in *Ca*Aga1, respectively [18]. The latter set of amino acid residues, lacking the key aromatic residues, could not constitute an effective sugar-binding cleft.

### 2.3. Recombinant CaAga1 Production, Purification, and Agarase Activity

**Figure 3:**
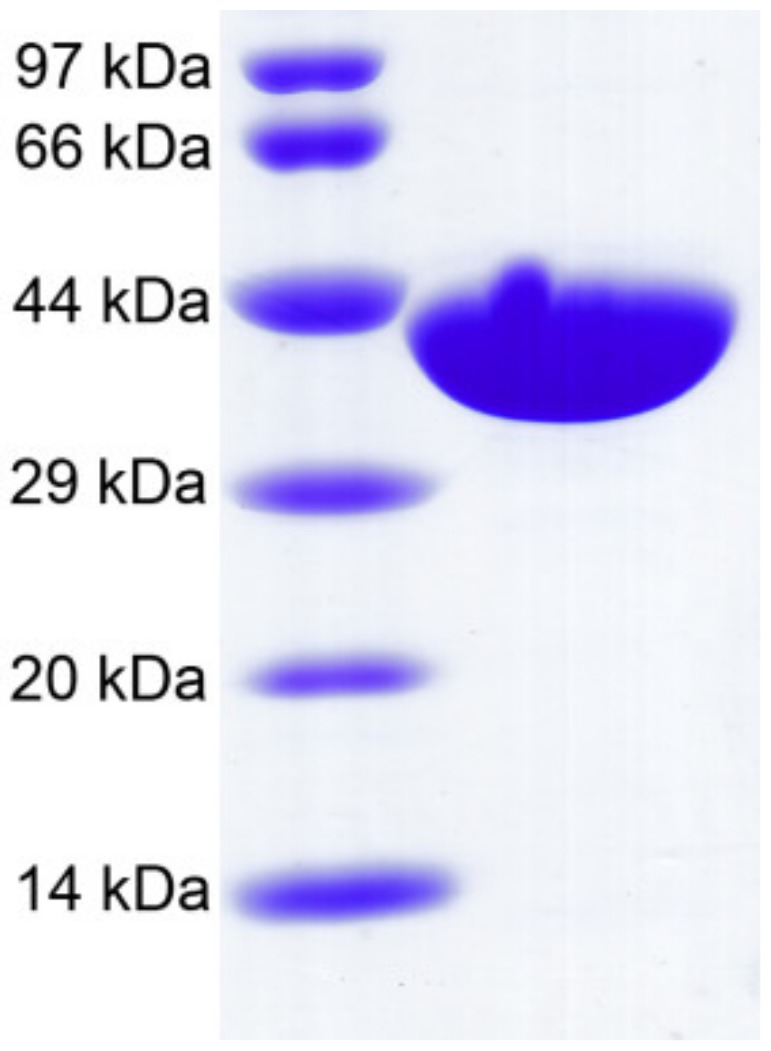
SDS-PAGE analysis of the purified recombinant *Ca*Aga1.

*Ca*Aga1 was produced in mature form (without signal peptide) by using the pET-28a expression vector. Thirty-six amino acids encoded by the expression vector were fused at the amino terminal of the protein. The recombinant *Ca*Aga1 contained 343 amino acids. The enzyme was purified by Ni^2+^ affinity chromatography. The final yielded enzyme displayed a single band, consistent with the calculated molecular mass of 39014.75 Da (Figure 3). The concentration of the final isolated recombinant *Ca*Aga1 was 2.85 mg/mL.

The purified *Ca*Aga1 were able to hydrolyze agarose, pure agar, and crude agars extracted from *Gracilaria lemaneiformis* and *Porphyra haitanensis*, with specific activity levels of 29.39, 20.20, 14.12, and 8.99 U/mg, respectively.

### 2.4 Biochemical Characterization of CaAga1

**Figure 4:**
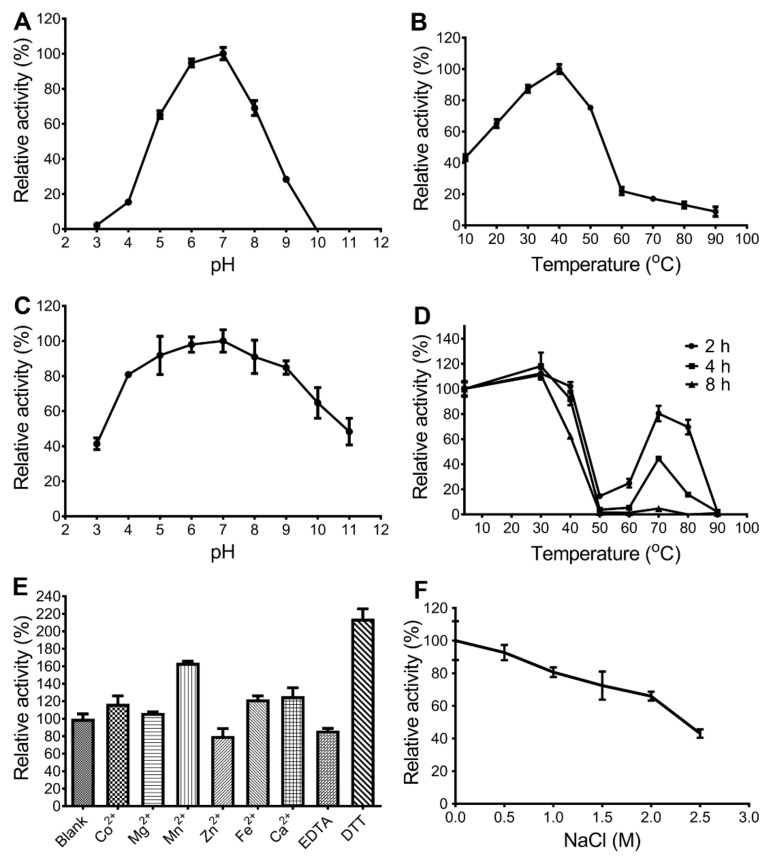
Effects of pH, temperature, and additives on the activity of *Ca*Aga1. Agarase activity was measured with 0.2% agarose as the substrate. (**A**) Optimal pH of *Ca*Aga1. The pH profile was measured at 40 °C and different pH ranges (3–8: citrate-phosphate buffer; 9–11: 50 mM glycine-NaOH buffer). (**B**) Optimal temperature of *Ca*Aga1. The temperature profile was assayed at temperatures ranging from 10 °C to 90 °C in a citrate-phosphate buffer (pH 7). (**C**) pH stability of *Ca*Aga1. The pH stability of *Ca*Aga1 was investigated by measuring the residual activity after incubating the enzyme in the buffers of different pH levels at 20 °C for 2 h. (**D**) Thermal stability of *Ca*Aga1. To assess the thermal stability, the enzymes were pre-incubated individually at different temperatures (10 °C to 90 °C) and then the activity was determined at 40 °C using enzymes that were collected at different times. (**E**) Effects of metal ions, chelating agent, and reducing agent on the activity of *Ca*Aga1. (**F**) Effect of NaCl on the activity of *Ca*Aga1. The values are shown as the percentages of the highest activity (for optimal pH and temperature determinations) or the percentages of the activity measured without pretreatment or additives (for determination of the enzyme stability and the effects of chemical reagents). The values for each point are presented as the average relative activity ± standard deviation (SD) for the three assays.

The effects of pH and temperature on the activity of *Ca*Aga1 were determined by measuring the relative activity with agarose as the substrate. The results showed that the optimal pH of *Ca*Aga1 was 7; the enzyme exhibited more than 65% of the maximum activity within the pH range of 5 to 8 (Figure 4A). *Ca*Aga1 showed maximum activity at 40 °C and maintained more than 75% of its maximum activity at 30 °C–50 °C (Figure 4B). *Ca*Aga1 kept approximately 43% of the maximum activity at 10 °C, indicating that it adapted to low temperatures.

*Ca*Aga1 exhibited outstanding pH stability and retained more than 40% residual activity after 2 h of incubation at pH levels ranging from 3 to 11 (Figure 4C). The enzyme was stable at 40 °C and reserved 90% residual activity after incubation at 40 °C for 4 h (Figure 4D). At medium-high temperatures (50 °C–60 °C), *Ca*Aga1 was thermolabile. The agarase activity severely declined after 2 h of incubation and was completely lost after 4 h of incubation at 50 °C and 60 °C. However, the enzyme showed good thermostability at high temperatures, that is, it maintained at least 80% activity after being kept at 70 °C for 2 h and more than 40% for 4 h (Figure 4D).

Among the tested metal ions, the agarase activity of *Ca*Aga1 was only inhibited in the presence of Zn^2+^. The other metal ions, including Co^2+^, Mg^2+^, Mn^2+^, Fe^2+^, and Ca^2+^, enhanced the activity at varying degrees (Figure 4E). Mn^2+^ showed the most significant enhancement effect (more than 60%). The chelating agent (ethylenediaminetetraacetic acid, EDTA) decreased the activity of *Ca*Aga1 by 20%. The reducing agent (dithiothreitol, DTT) increased the activity of *Ca*Aga1 by 110%. *Ca*Aga1 maintained approximately 70% activity in the presence of 2 M NaCl (Figure 4F).

With agarose as substrate and under the optimal reaction condition (pH 7 and 40 °C), the *K_m_*, *V_max_*, and *k_cat_* values of *Ca*Aga1 were found to be 1.19 mg/mL, 36.21 U/mg, and 24.03 s^−1^, respectively (Table 1).

### 2.5 Hydrolytic Products of CaAga1

**Figure 5:**
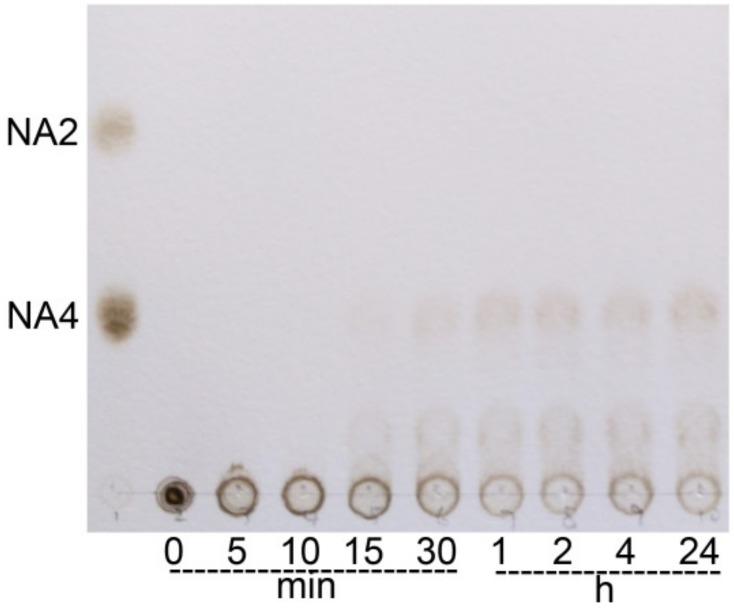
Thin layer chromatography analysis of *Ca*Aga1-catalyzed hydrolysates of agarose. NAOS standards—NA2 (neoagarobiose) and NA4 (neoagarotetraose).

The hydrolysate profile of *Ca*Aga1 was investigated through thin layer chromatography (TLC) with agarose as the substrate. No NA2 was detected, suggesting that *Ca*Aga1 is an endo-type agarase. The analysis of hydrolysates from time-course hydrolysis indicated the formation of NAOSs (mainly NA4 and NA6) after 15 min (Figure 5). The hydrolysis was terminated in about 1 h. The amount and length of NAOSs did not change from 1 h to 24 h. Therefore, the end products were confirmed to be NA4 and NA6.

## 3. Discussion

The agarolytic bacterium *C. algicola* DSM 14237 was isolated from the Eastern Antarctic coastal zone. The bacterium produces a wide range of cold-adapted glycoside hydrolases, which have great potential for industrial and biotechnical applications. This study reported the biochemical characterization of a putative agarase, *Ca*Aga1, in the genome of *C. algicola* DSM 14237. *Ca*Aga1 is composed of a 21-residue signal peptide and a catalytic domain belonging to the GH16 family. The agarase shows the highest identity (76%) to β-agarase from *C. omnivescoria* W5C. The recombinant *Ca*Aga1 was active toward agarose, pure agar, and crude agars from marine red algae. With agarose as the substrate, *Ca*Aga1 had a *K_m_* value of 1.19 mg/mL which is lower than that reported for most of β-agarases (Table 1).

*Ca*Aga1 showed maximum activity at a temperature (40 °C) similar to most of the reported agarases (30 °C–50 °C) [2,3]. However, *Ca*Aga1 retained an important percentage of enzymatic activity at low temperatures (approximately 43% at 10 °C), implying the adaptation of the enzyme to cold conditions. The agarases that shared similar properties to *Ca*Aga1 were characterized as AgaJ5 (GH86, from *Gayadomonas joobiniege* G7), AgaJ9 (GH39, from *G. joobiniege* G7), and Aga21 (from *Pseudoalteromonas* sp. NJ21), which had 40% activity at 10 °C [19], 80% activity at 5 °C [20], and 80% activity at 5 °C [21], respectively (Table 1). The cold-adapted feature enables *Ca*Aga1 to act in energy saving conditions (lowering the reaction temperature) and is of special interest for industrial applications.

Generally, stability is a required property for the biotechnological utilization of enzymes because of the harsh conditions that are mostly found in various industrial processes. The most impressive biochemical characteristics of *Ca*Aga1 are its excellent stability, including pH stability, thermal stability, and salt tolerance. *Ca*Aga1 was halotolerant, which is the general property of enzymes derived from the marine environment. The pH range that *Ca*Aga1 can tolerate was broader than that of the reported pH-stable agarases (Figure 4C). AgaB34 from *Agarivorans albus* YKW-34 showed comparable pH stability to that of *Ca*Aga1; the agarase retained about 40% activity at pH 4–11 [22]. AgaB from *Flammeovirga* sp. SJP92 was only stable under neutral and alkaline conditions (>pH 5) [23]. The characterized agarases were thermolabile due to the fact that agarases generally originated from a marine environment. Most agarases were stable at temperatures up to 45 °C [3] (Table 1). Only few agarases were stable at temperatures more than 50 °C, and they were Aga16B from *Saccharophagus degradans* 2-40^T^ (100%, at 50 °C for 2 h) [15], Aga4436 from *Flammeovirga* Sp. OC4 (35%, at 50 °C for 144 h) [24], AgaB34 from *A. albus* YKW-34 (>80%, at 50 °C for 1 h) [22], agarase-fst from *Thalassospira profundimonas* (70%, at 50 °C for 1 h) [25], AgaP4383 from *Flammeovirga pacifica* WPAGA1 (100%, at 50 °C for 10 h) [26], AgaP from *Pseudoalteromonas* sp. AG4 (80%, at 55 °C for 1 h) [27], AgaA7 from deep-sea *Microbulbifer* (50%, at 50 °C for 8 h) [28], and AgaM1 from the environmental DNA of mangrove sediments (84%, at 50 °C for 3 h) [29] (Table 1). Compared with these thermostable agarases, *Ca*Aga1 displayed an unusual temperature profile for thermostability. *Ca*Aga1 was stable at two temperature ranges, the low (40 °C and below) and high (around 70 °C) temperature ranges, but unstable at temperatures between these ranges (Figure 4D). Similar thermal characteristic were observed for AgaA from *Agarivorans* sp. LQ48 [30] and SSG-1a from *Paenibacillus* sp. SSG-1 [31]. However, no description and explanation for the phenomenon were addressed in the related reports [30,31]. The likely explanation is that a more ordered structure (secondary and tertiary), different from the original, was adopted by *Ca*Aga1. The conformation change was induced by high temperatures (70 °C) but could not be facilitated by medium-high temperatures (50 °C–60 °C). Such a thermodynamic stability proposed for *Ca*Aga1 needs to be proved using differential scanning fluorimetry or molecular dynamics simulation in following studies.

Many β-agarases from the GH16 family, including *Ca*Aga1, could hydrolyze the agarose chain into NA4 and NA6, and other enzymes produced shorter NAOSs, namely, NA2 as the end products [8]. The generation of NA2 depends on whether or not β-agarases can cleave NA6. To speculate the determinant amino acids of the degradation pattern, we compared the amino acids involved in agarose chain binding in the substrate-binding cleft of GH16 agarases with the end hydrolysis products of the enzymes. The NA2-producing agarases included N3-1 from *Microbulbifer* sp. BN3 [32], YM01-1 from marine bacterium *C. agarivorans* YM01 [33], AgaH71 from *Pseudoalteromonas hodoensis* [34], AgaG1 from *Alteromonas* sp. GNUM-1 [35], and *Zg*AgaA and *Zg*AgaB from *Z. galactanivorans* Dsij [36,37] (Table 1). The most notable amino acid difference between the NA2-producing agarases and the other GH16 agarases was located at the +4 subsite. The +4 subsite was generally composed of two consecutive aromatic amino acid residues, tryptophan and tyrosine or phenylalanine (WY, or WF) (Trp197 and Tyr198 in *Ca*Aga1). These amino acids appeared to be WF (in N3-1, AgaH71, and *Zg*AgaB), RY (in YM01-1), EY (in AgaG1), and YF (in *Zg*AgaA) (Figure 1 and Figure 2). Structural studies proposed that the substrate-binding cleft of GH16 agarases could maximally accommodate eight sugar residues [8,18]. Four or five Trp residues in the cleft were proposed to be critical for sugar ring binding [18]. Among the Trp residues, only Trp at −3, +2, and +4 subsites could form a hydrophobic stacking interaction with D-galactose (Figure 2). Therefore, we proposed a structural explanation to the cleavage pattern of GH16 β-agarases. In agarases, such as *Ca*Aga1, the NA6 molecule prefers to bind to a region (−2 to +4) that contains two closer Trp-containing subsites (+2 and +4) due to the presence of the Trp at the +4 subsite. The catalytic reaction will not occur in such a positional binding because the main binding affinity between the enzyme and substrate is located in the aglycone subsites. When the Trp residue at the +4 subsite is missing, NA6 will bind to the −4 to +2 subsites, forming stacking interactions at the −3/+2 Trp. Accordingly, NA2 and NA4 are produced. Another amino acid (Tyr198 in *Ca*Aga1) at the +4 subsite strengthens the binding affinity at the +4 subsite by providing an additional hydrogen bond (Figure 2). This hypothesis explains the lower activity of *Zg*AgaA toward NA6 than *Zg*AgaB, although they both produced NA2 [36]. Identifying the precise and additional determinants of the cleavage pattern for GH16 agarases requires the determination of the structures of GH16 β-agarases in complex with NAOSs of different lengths.

## 4. Materials and Methods

### 4.1. Materials

Standard NAOSs (neoagarobiose, NA2; neoagarotetraose, NA4) were purchased from Qingdao BZ-Oligo Co., Ltd. (Qingdao, China). Commercial agar (Cat. No. A1296) and agarose (Cat. No. A9539) were purchased from Sigma-Aldrich (Shanghai, China). Red algae (*G. lemaneiformis* and *P. haitanensis*) were purchased from Putian, Fujian province, China.

### 4.2. Amino Acid Sequence Analysis

The signal peptide was predicted using the SignalP-5.0 Server [38]. The protein sequence analysis was carried out using BLASTp search (https://blast.ncbi.nlm.nih.gov/Blast.cgi). The amino acid sequence alignment of agarases (GH16 domains) was conducted using Clustal Omega (https://www.ebi.ac.uk/Tools/msa/clustalo/). The three-dimensional structural model of *Ca*Aga1 was generated by homology modeling using SwissModel (http://swissmodel.expasy.org/) [39]. The structural figures were prepared using PyMOL (Schrödinger LLC, Cambridge, MA, USA).

### 4.3. Cloning and Expression of Agarases

The codon optimized DNA fragments (without the signal peptide sequence) encoding the putative agarase (*Ca*Aga1) from *C. algicola* DSM 14237 were synthesized by GENEWIZ Suzhou Company (Suzhou, China) according to the annotated genome sequence deposited at GenBank (Accession number: NC_014934.1). The synthesized *Ca*Aga1 gene was on the pUC57-simple vector (pUC57-*Ca*Aga1) with *BamH*1 and *Xho*1 restriction sites added to its 5’- and 3’-ends, respectively. The DNA fragments of *Ca*Aga1 (obtained by double digestion of pUC57-*Ca*Aga1 with *Bam*H1 and *Xho*1) were sub-cloned into the expression vector pET-28a (Invitrogen, Shanghai, China). The resulting recombinant plasmids (pET-28a-*Ca*Aga1) were transformed into *Escherichia coli* BL21 Rosetta (DE3) competent cells (Thermo Fisher Scientific, Shanghai, China).

The recombinant protein production and purification were performed as described previously by Han et al. [40]. In brief, the overnight culture (5 mL) of the transformed cells was prepared to inoculate 1 L of ZYM 5052 auto-induction medium supplemented with 34 µg/mL chloramphenicol and 100 µg/mL kanamycin in a 2 L shake flask [41]. The culture was incubated at 37 °C for 4 h with shaking (250 rpm), and then the cells were grown at 20 °C for 20 h with the same shaking condition. The cell harvest was made by centrifugation (5000 rpm for 30 min at 4 °C). The cell pellet was re-suspended in 40 mL of lysis buffer (500 mM NaCl, 20 mM Na_2_HPO_4_, 20 mM imidazole, pH 7.4) and disrupted using a high-pressure homogenizer. The supernatant of the cell lysate (filtered over a 0.45-μm filter) was loaded to a 5 mL HisTrap column (GE Healthcare, Beijing, China). The recombinant protein was eluted from the column with an elution buffer (500 mM NaCl, 20 mM Na_2_HPO_4_, 500 mM imidazole, pH 7.4) and was analyzed on 12% sodium dodecylsulfate polyacrylamide gel electrophoresis (SDS-PAGE). The relative pure eluted fractions were pooled and dialyzed into a 25 mM Tris-HCl buffer (pH 8.0). The protein concentration was determined using the Bradford method with bovine serum albumin as the standard.

### 4.4. Preparation of Crude Agar from Red Algae

In brief, 10 g of dry red algae (*G. lemaneiformis* or *P. haitanensis*) were washed with fresh water and soaked in 100 mL of an alkali solution (5% sodium hydroxide) at 80 °C for 2 h. The alkali solution was discarded, and the red algae were washed three times with fresh water. The red algae were then soaked in 200 mL of distilled water and placed in an autoclave for 1 h (121 °C). The hot seaweed extract was filtered using cheesecloth (eight layers). The crude agar was obtained after removing the water in the extract through freezing and thawing. The crude agar was further dried in an oven at 60 °C overnight.

### 4.5. Standard Agarase Assay

The activity of the recombinant *Ca*Aga1 was measured using the dinitrosalicylic acid (DNS) method [42]. The standard reaction mixture contained 100 μL of citrate-phosphate buffer (pH 7), 100 μL of 0.2% (*w*/*v*) melted substrate, and 100 μL of the diluted *Ca*Aga1 (approximately 20 µg/mL). After 20 min of incubation in a water bath (40 °C), the reaction mixtures were added with 300 μL of DNS. The color was developed by heating the mixture in boiling water for 5 min. The reducing sugar released was quantified by recording the absorbance at 520 nm. Galactose was used to prepare a standard curve for evaluating the amount of reducing sugars. One unit (U) of agarase activity was defined as the amount of enzyme (mg) required to release 1 μmol D-galactose per minute. All assays were conducted in triplicate.

### 4.6. Biochemical Characterization

The biochemical characteristics of *Ca*Aga1 were investigated with 0.2% agarose as substrate. The optimal pH of *Ca*Aga1 was determined at 40 °C and pH 3–11. A citrate-phosphate buffer (pH 3.0–8.0) and glycine-NaOH buffer (50 mM Tris-HCl, pH 9.0–11.0) were used to prepare the pH gradients. The optimal temperature of *Ca*Aga1 was assayed at pH 7 and 10 °C to 90 °C. The pH tolerance profile of *Ca*Aga1 was estimated by measuring residual agarase activity under the optimal condition after 2 h of incubation at 20 °C over a pH range of 3–11. In specific, for the individual assay, 1 µl of purified enzyme was diluted 20 times with buffers of different pH values for incubation, and the enzymes after incubation (20 µl) were subjected to an agarase assay (at pH 7). The thermal stability of *Ca*Aga1 was determined by testing the residual agarase activity under the optimal condition after incubation at different temperatures for 2, 4, or 8 h. All assays were performed in triplicate.

Metal ions (final concentration of 5 mM), chelating agent (EDTA, final concentration 10 mM), reducing agent (DTT, final concentration 10 mM), or NaCl (final concentration 0.5 to 2.5 M) were added to the standard reaction mixtures to assess their effects on *Ca*Aga1 activity. The agarase activity measured in the absence of the above additives was taken as 100%. Each assay was performed with at least three replicates.

The initial velocities of *Ca*Aga1 at different agarose concentrations (0.5–5 mg/mL) were determined by measuring the agarase activity at pH 7.0 and 40 °C for 10 min. The *K_m_* and *V_max_* values were obtained from the Lineweaver-Burk plot constructed from the initial velocity data. The *k_cat_* value was calculated from the *V_max_* value considering the amount of enzyme (0.5 µg) used in the individual reaction.

### 4.7 Analysis of Hydrolysis Products

The hydrolysis pattern of *Ca*Aga1 was investigated by TLC with agarose as the substrate. The hydrolysis products were obtained through agarase reactions under the optimal condition. The samples were collected at different time points (0 min, 5 min, 10 min, 15 min, 30 min, 1 h, 2 h, 4 h, and 24 h). The samples were analyzed on a Silica Gel 60 plate (Merck, Darmstat, Germany) by using n-butanol/acetic acid/water (2:1:1; *v*/*v*/*v*) as the solvent. The oligosaccharide bands were visualized by spraying the plate with a solution containing 5% H_2_SO_4_ (*v*/*v*, in methanol) and heating the plate at 115 °C for 5 min. Pure NA2 and NA4 were used as the standards.

## Figures and Tables

**Table 1:** Biochemical properties of characterized agarases.

Agarase	Glycoside Hydrolase Family	Optimal Temperature	Optimal pH	Thermal Stability (Retaining Activity)	pH Stability (Retaining Activity)	Product	Kinetic Parameter	Reference and Accession Number
*Ca*Aga1 (*Cellulophaga algicola* DSM 14237)	16	40	7	90%, 40 °C, 4 h; 80%, 70 °C, 2 h	80%, pH 4–9, 20 °C, 2 h	NA4, NA6	*K_m_*: 1.19 mg/mL, *V_max_*: 36.21 U/mg	This study WP_013551224.1
N3-1 (Microbulbifer sp. BN3)	16	50	6	40%, 30–50 °C, 30 min	80%, pH 4–9, room temperature, 1 h	NA2, NA4		[32] AXY54922.1
AgaM1 (environmental DNA ofmangrove sediments)	16	50	7	80%, 40–50 °C, 3 h	80%, pH 5–10, 50 °C, 3 h	NA4, NA6	*K_m_*: 1.82 mg/mL, *V_max_*: 357.14 U/mg	[29] AYA70425.1
YM01-1 (*Catenovulum agarivorans* YM01)	16	50	7	<10%, 70 °C, 1 h	80%, pH 6–9, 4 °C, 12 h	NA2	*K_m_*: 8.69 mg/mL, *V_max_*: 4350 U/mg	[33] ATI96843.1
Aga2 (*Cellulophaga omnivescoria* W5C)	16	45	8			NA4, NA6	*K_m_*: 2.59 mg/mL, *V_max_*: 275.48 U/mg	[17] ATI14840.1
Aga16B (*Saccharophagus degradans* 2-40^T^)	16	40–60	7.5	100%, 50 °C, 2 h		NA4, NA6		[15] Q21LJ2
AgaB (*Flammeovirga* sp. SJP92)	16	45	8	60%, 55 °C, 30 min	50%, pH 6–9, room temperature, 1 h	NA4, NA6	*K_m_*: 3.99 mg/mL, *V_max_*: 700 U/mg	[23] ANN44251.1
Aga4436 (*Flammeovirga* sp. OC4)	16	50–55	6	80%, 40 °C, 144 h	70%, pH 3–10, 50 °C, 1 h	NA4, NA6		[24] AJW82062.1.
Aga21 (*Pseudoalteromonas* sp. NJ21)	16	30	8	20%, 40 °C, 1 h		NA2		[21]
AgaH71 (*Pseudoalteromonas hodoensis*)	16	45	6	90%, 45 °C, 1 h		NA2, NA4, NA6	*K_m_*: 28.33 mg/mL, *V_max_*: 88.25 U/mg	[34] AIF29515.1
AgaG1 (*Alteromonas* sp. GNUM-1)	16	40	7	70%, 45 °C, 30 min		NA2	*K_m_*: 3.74 mg/mL, *V_max_*: 23.8 U/mg	[35] AGW43026.1
AgaA (*Agarivorans* sp. LQ48)	16	40	7	95%, 40 °C, 1 h	95%, pH 3–11, 20 °C, 1 h	NA4, NA6	*K_m_*: 43.9 mg/mL, *V_max_*: 909.1 U/mg	[30] ACM50513.1
AgaP (*Pseudoalteromonas* sp. AG4)	16	55	5.5	75%, 55 °C, 1 h		NA4		[27] ADD60418.1
AgaB34 (*Agarivorans albus* YKW-34)	16	30	7	80%, 50 °C, 1 h	70%, pH 5–9, 40 °C, 1 h	NA4	*K_m_*: 0.24 mg/mL, *V_max_*: 50 U/mg	[22] ABW77762.1
ZgAgaA (*Zobellia galactanivorans*)	16		6			NA2 (minor), NA4, NA6	*K_m_*: 2 mg/mL, *k_cat_*: 150 s^−1^	[37] AAF21820.1
ZgAgaB (*Zobellia galactanivorans*)	16		7			NA2, NA4,	*K_m_*: 1 mg/mL, *k_cat_*: 100 s^-1^	[37] AAF21821.1
AgaA7 (deep-sea *Microbulbifer*)	16	50	7	50%, 50 °C, 502 min	50%, pH 3.5–9.5, 40 °C, 30 min	NA4		[28] BAC99022.1
AgaJ9 (*Gayadomonas joobiniege* G7)	39	25	5	<20%, 40 °C, 30 min		NA2, NA4,	*K_m_*: 1.43 mg/mL, *V_max_*: 10.7 U/mg	[20] WP_017446561.1
AgaJ5 (*Gayadomonas joobiniege* G7)	86	30	4.5			NA6	*K_m_*: 8.9 mg/mL, *V_max_*: 188.6 U/mg	[19] WP_017446675.1
AgaP4383 (*Flammeovirga pacifica* WPAGA1)	86	50	9	100%, 50 °C, 10 h	90%, pH 5–10, 30 °C, 24 h	NA4, NA6	*K_m_*: 8.53 mg/mL, *V_max_*: 1.2 U mg	[26] AIA22721.1
Agarase-fst (*Thalassospira profundimaris* fst-13007)		45	8	70%, 50 °C, 1 h	80%, pH 5–8, 45 °C, 1 h	NA2, NA4, NA6		[25]
SSG-1a (Paenibacillus sp. SSG-1)		50	6	95%, 40 °C, 1 h		NA8		[31]
